# Effects of acute warming on cardiac and myotomal sarco(endo)plasmic reticulum ATPase (SERCA) of thermally acclimated brown trout (*Salmo trutta*)

**DOI:** 10.1007/s00360-020-01313-1

**Published:** 2020-09-26

**Authors:** Matti Vornanen

**Affiliations:** Department of Environmental and Biological Sciences, University of Eastern Finland, P.O. Box 111, 80101 Joensuu, Finland

**Keywords:** Temperature tolerance, Fish heart, Calcium management, Heart rate

## Abstract

At high temperatures, ventricular beating rate collapses and depresses cardiac output in fish. The role of sarco(endo)plasmic reticulum Ca^2+^-ATPase (SERCA) in thermal tolerance of ventricular function was examined in brown trout (*Salmo trutta*) by measuring heart SERCA and comparing it to that of the dorsolateral myotomal muscle. Activity of SERCA was measured from crude homogenates of cold-acclimated (+ 3 °C, c.a.) and warm-acclimated (+ 13 °C, w.a.) brown trout as cyclopiazonic acid (20 µM) sensitive Ca^2+^-ATPase between + 3 and + 33 °C. Activity of the heart SERCA was significantly higher in c.a. than w.a. trout and increased strongly between + 3 and + 23 °C with linear Arrhenius plots but started to plateau between + 23 and + 33 °C in both acclimation groups. The rate of thermal inactivation of the heart SERCA at + 35 °C was similar in c.a. and w.a. fish. Activity of the muscle SERCA was less temperature dependent and more heat resistant than that of the heart SERCA and showed linear Arrhenius plots between + 3 and + 33 °C in both c.a. and w.a. fish. SERCA activity of the c.a. muscle was slightly higher than that of w.a. muscle. The rate of thermal inactivation at + 40 °C was similar for both c.a. and w.a. muscle SERCA at + 40 °C. Although the heart SERCA is more sensitive to high temperatures than the muscle SERCA, it is unlikely to be a limiting factor for heart rate, because its heat tolerance, unlike that of the ventricular beating rate, was not changed by temperature acclimation.

## Introduction

Sarcoplasmic reticulum (SR) functions as an intracellular Ca^2+^ store in striated muscle cells and regulates the rate of rise and fall of intracellular Ca^2+^-transient in excitation–contraction (e-c) coupling (Fabiato 1983; Rossi and Dirksen 2006; Periasamy and Kalyanasundaram 2007). In skeletal muscle fibres, the amplitude of intracellular Ca^2+^ transient is almost exclusively dependent on SR Ca^2+^ release via ryanodine receptors, which are triggered to open by sarcolemmal depolarization in the voltage-induced Ca^2+^ release process (Melzer et al. 1995). Contraction of cardiac myocytes depends on both sarcolemmal Ca^2+^ influx and SR Ca^2+^ release. In mammalian cardiac myocytes, a small sarcolemmal Ca^2+^ entry triggers a much larger Ca^2+^ release from the SR in the Ca^2+^-induced Ca^2+^ release process (Fabiato 1983). In cardiac myocytes of many fish species, SR Ca^2+^ release plays a smaller role in triggering contraction in comparison to sarcolemmal Ca^2+^ influx, which provides a major part of contractile Ca^2+^ via L-type Ca^2+^ channels and Na^+^–Ca^2+^ exchange (Tibbits et al. 1991; Vornanen et al. 2002). However, in the highly active and partially endothermic tuna fish, which have higher heart rates, SR may play a more significant role in cardiac e-c coupling (Keen et al. 1992; Shiels et al. 1999, 2015). Furthermore, acclimation to cold increases the role of SR Ca^2+^ management in e-c coupling of the cold-active fish species (Keen et al. 1994; Aho and Vornanen 1999).

In striated muscle cells, the lumenal SR Ca^2+^ content must be replenished after each contraction to maintain proper efficacy of SR Ca^2+^ release, which is critically dependent on SR Ca^2+^ load (Bassani et al. 1995; Eisner et al. 2013). Sequestration of cytosolic Ca^2+^ back into the SR is accomplished by the sarco(endo)plasmic reticulum Ca^2+^-ATPase or SERCA. Three SERCA genes (ATP2A1-3) have been found in vertebrates. SERCA2 is the major cardiac isoform, while SERCA1 is the skeletal isoform. The heart gene codes for three functionally different splice variants, SERCA2a/b/c, and the muscle gene two SERCA1a/b splice variants (Thomas and Hanley 1994; Londraville et al. 2000; Wuytack et al. 2002; Periasamy and Kalyanasundaram 2007).

Although the expression and activity of the SERCA2 is known to be changed by temperature acclimation in rainbow trout (*Oncorhynchus mykiss*) and burbot (*Lota lota*) hearts (Aho and Vornanen 1998; Korajoki and Vornanen 2012, 2013), our understanding of high temperature tolerance of the heart SERCA in fish is still incomplete. It is important to understand the significance of temperature tolerance of the heart SERCA in cardiac e-c coupling, because at critically high temperatures, cardiac output collapses in fish (Stevens et al. 1972). The heat-induced failure of cardiac output is almost solely due to depression of ventricular beating rate, since stroke volume is largely independent of temperature (Randall 1968; Steinhausen et al. 2008; Mendonca and Gamperl 2010; Ekström et al. 2014). To fully understand the temperature-induced heart failure in fish, the mechanistic explanation for the deterioration of ventricular contractions needs to be known. When heart rate increases, there is less time for each cardiac cycle and, therefore, Ca^2+^ removal from the cytosol must be faster to leave enough time for diastolic filling of the ventricle. Indeed, heart rate correlates strongly with SERCA activity in various vertebrate species and during ontogenetic development of mammals (Fabiato 1982; Hamilton and Ianuzzo 1991; Vornanen 1992; Su et al. 2003; Vangheluwe et al. 2005; Rosati et al. 2008). Although the role of SR Ca^2+^ cycling in fish hearts is less than in endothermic hearts, SR Ca^2+^ uptake might become limiting for ventricular beating rate at high temperatures if its activity collapses. Furthermore, SR Ca^2+^ release may be involved in the rate generator of the sinoatrial pacemaker (Monfredi et al. 2013) and, therefore, thermal failure of the SERCA could compromise pacemaker rate at high temperatures. Experiments on the ventricular SERCA of the tuna fish and other scombrid species suggest that ATPase activity and Ca^2+^ uptake by SERCA is resistant to temperatures which the fish are likely to meet in their habitat (Landeira-Fernandez et al. 2004; Castilho et al. 2007). Similarly, SERCA of the + 21 °C-acclimated rainbow trout (*Oncorhyncus mykiss*) ventricle maintains high activity between + 5 °C and + 30 °C (Da Silva et al. 2011).

If heart function is particularly sensitive to high temperature relative to other body functions and, therefore, the limiting factor for whole animal thermal tolerance (Farrell 2009), and if SERCA activity is the limiting factor for the contraction rate of the ventricle, the following hypotheses should be valid: (1) SERCA activity of the heart is more sensitive to high temperatures than the activity of the muscle SERCA. (2) In thermal acclimation, temperature tolerance of the heart SERCA changes in similar manner to temperature tolerance of the ventricular beating rate i.e. the fish acclimated to warm have a more heat-resistant SERCA than the fish acclimated to cold (Bowler 1981). Previous studies have shown that in brown trout acclimated to cold ventricular beating rate collapses at significantly lower temperatures than in brown trout acclimated to warm (Vornanen et al. 2014; Haverinen et al. 2017). Therefore, the above hypotheses were tested with the heart SERCA of thermally acclimated brown trout and temperature responses of the heart SERCA were compared to those of the dorsolateral myotomal muscle. It is shown that the heart SERCA is less heat tolerant than the muscle SERCA, but temperature acclimation does not have any effect on heat tolerance of either heart or muscle SERCA.

## Materials and methods

### Animals

Brown trout (*Salmo trutta*) were kindly donated by the local fish-farm (Kontiolahti, Finland). In the animal facilities of the University of Eastern Finland (Joensuu), the animals were randomly divided in two refrigerated metal tanks (500 L) filled with ground water at the rearing temperature of the fish-farm. Thereafter, the temperature was changed at the rate of about 1 °C day^−1^ until acclimation temperatures of + 3 °C (cold acclimated, c.a.; 104.37 ± 4.67 g, 22.30 ± 0.37 cm, *n* = 29) or + 13 °C (warm acclimated, w.a. 90.45 ± 4.77 g, 22.25 ± 0.38 cm, *n* = 30) were attained (Computec, Joensuu Finland). Animals were used in experiments not earlier than after 3 weeks of accommodation at the acclimation temperatures. Aerated water was constantly flowing through the tanks at the rate of about 200 L day^−1^ and temperature of the water was under constant computer control. Fish were fed trout feed (Ewos, Turku, Finland) five times per week. The experiments were authorized by the national animal experimental board in Finland (permissions STH252A and ESAVI/8877/2019).

### Determination of SERCA activity

Comparative studies in mammalian species have shown that in the isolation process of cardiac microsomes by differential centrifugation the yield of SR protein varies from species to species and does not correspond to the actual SR content of myocytes, or the SR enzymes are inactivated during the long isolation procedure (Feher and LeBolt 1990; Hamilton and Ianuzzo 1991). In addition, it is difficult to get enough SR microsomes from hearts of fish species, where the SR is less well-developed (Landeira-Fernandez et al. 2004). To avoid these problems, we used unfractionated tissue homogenates for measurement of SERCA activity, and validated the method for use in both cardiac and muscle preparations. To this end, the fish were killed by a stunning stroke to the head and bleeding. The whole ventricle of the heart and a small piece of the dorsolateral myotomal muscle were used for SERCA determination (From here on, we refer to these preparations as heart and muscle SERCA, respectively). Tissue samples were weighed and placed in ice-cold homogenization buffer if SERCA was determined in the same day, or snap frozen in liquid nitrogen and stored at − 50 °C if determined later. The homogenization buffer contained (in mM) sucrose 200, EDTA 1, NaN_3_ 5 and Hepes 40 (pH 7.2) at room temperature. Tissue samples were homogenized in 30 volumes of ice-cold buffer for 30 s in a Potter–Elvehjem type glass/teflon (muscle) or a glass/glass (heart) homogenizer driven by a Heidolph stirrer at 2000 rounds min^−1^. The unfractionated muscle/heart homogenate was immediately used for SERCA determinations.

SERCA activity was measured as a cyclopiazonic acid (CPA) or thapsigargin (TG) sensitive ATPase activity in the total volume of 1 mL of the incubation medium (Table 1). The medium included inhibitors of mitochondrial ATPase (5 mM sodium azide, NaN_3_) and Na^+^/K^+^-ATPase (0.01 mM ouabain), and Triton X-100 (0.005% v/v) to permeabilize membrane vesicles and thereby prevent accumulation of Ca^2+^ within the membrane vesicles. EGTA (10 mM) was used to set the free Ca^2+^ concentration to support the maximum activity of the SERCA. In the presence of the total Ca^2+^ concentration of 1 mM, the free Ca^2+^ concentration was calculated to be 8.0 µM at pH 7.2 (+ 20 °C) (Webmaxc Standard; https://somapp.ucdmc.ucdavis.edu/harmacology/bers/maxchelator/webmaxc/webmaxcS.htm). pH of the incubation buffer was allowed to change in alpha-stat manner resulting in the free Ca^2+^ concentration between 5.3 µM (+ 3 °C) and 10.5 µM (+ 33 °C) at different temperatures. Since SERCA activity is optimal in the range of 5 and 10 µM free Ca^2+^ (Tupling et al. 2004; Gorski et al. 2013; Bidwell and Kranias 2016), the small changes in free Ca^2+^ concentration will not have any effect on the maximal SERCA activity. For SERCA determination, 0.9 ml incubation buffer was pipetted in 10 mL test tubes and the tubes were left to equilibrate at each test temperature for 5 min before the reaction was started by addition of 0.1 mL of the homogenate containing 25–50 mg of tissue. The reaction was stopped with 2 mL of a solution obtained by mixing 1% (NH_4_)_2_Mo_7_O_24_ 4H_2_O in 1.8 M H_2_SO_4_ with 1% Lubrol W (prepared daily in distilled water) in 1:1 (Atkinson et al. 1973). The incubation times for + 3, + 13, + 23 and + 33 °C were 60, 30, 15 and 10 min, respectively, to prevent the depletion of ATP. The liberated inorganic phosphate was immediately determined at the wavelength of 390 nm (Atkinson et al. 1973). *Q*_10_ value was calculated using the equation $Q_{10} = \left( {\frac{R2}{{R1}}} \right)^{{10^\circ {\text{C}}/\left( {T2 - T1} \right)}}$, where *R*1 and *R*2 are ATPase activities at the temperatures *T*1 and *T*2. Activation energy (*E*_a_) of the SERCA was calculated from the slope of the Arrhenius plot and gas constant (*R*): $Q_{10} = {\text{slope}}\left( K \right) \cdot 8.314\left( {{\text{J}}\,{\text{K}}^{ - 1} \,{\text{mol}}^{ - 1} } \right)$.

**Table 1:** The composition of the incubation medium of SERCA

Compound	Concentration (mM)
Hepes	20, pH 7.2
KCl	200
MgATP	5
MgCl_2_	10
CaCl_2_ · 6H_2_O	1
EGTA · 4K^+^	10
Ouabain	0.010
NaN_3_	5
K_2_Oxalate	10
CPA	0.020/0.000^a^
Triton X-100	0.005%

^a^SERCA activity was obtained as a difference in the rate of ATP hydrolysis in the absence and presence of CPA

### Thermal inactivation of SERCA

The rate of thermal inactivation of heart and muscle SERCA was measured at + 35 °C and + 40 °C, respectively. Preliminary experiments showed that the muscle SERCA does not inactivate at + 35 °C, and therefore, a higher temperature (+ 40 °C) was required for muscle homogenates. Homogenates were incubated at + 35 °C and + 40 °C for 0–60 min after which the SERCA activity was measured at + 25 °C for 15 min. Activity of SERCA was plotted as a function of the preincubation time and fitted to a monoexponential equation $f=y0+{a}^{(-b\cdot t)}$, where *a* is the activity explained by the exponent, *b* is the slope of the line (1/*b* = time constant, *τ*), *t* is time (min) and *y*0 is the unexplained part of the activity (about 10% of the total activity).

#### Statistics

The results are given as means ± SEM. The pH dependence of SERCA activity between heart and muscle SERCA, inhibition of SERCA by different concentrations of CPA and TG and temperature dependence of SERCA activity between acclimation groups were all compared by using two-way ANOVA (IBM SPSS software version 25.0). The rate of heat inactivation of SERCA was measured as the inactivation time constant (*τ*), which was compared between c.a. and w.a. fish using a non-parametric Mann–Whitney test (SigmaPlot 13). A *p* value of 0.05 was regarded as the criterion for statistical significance.

## Results

### pH dependence of SERCA

**Fig. 1:**
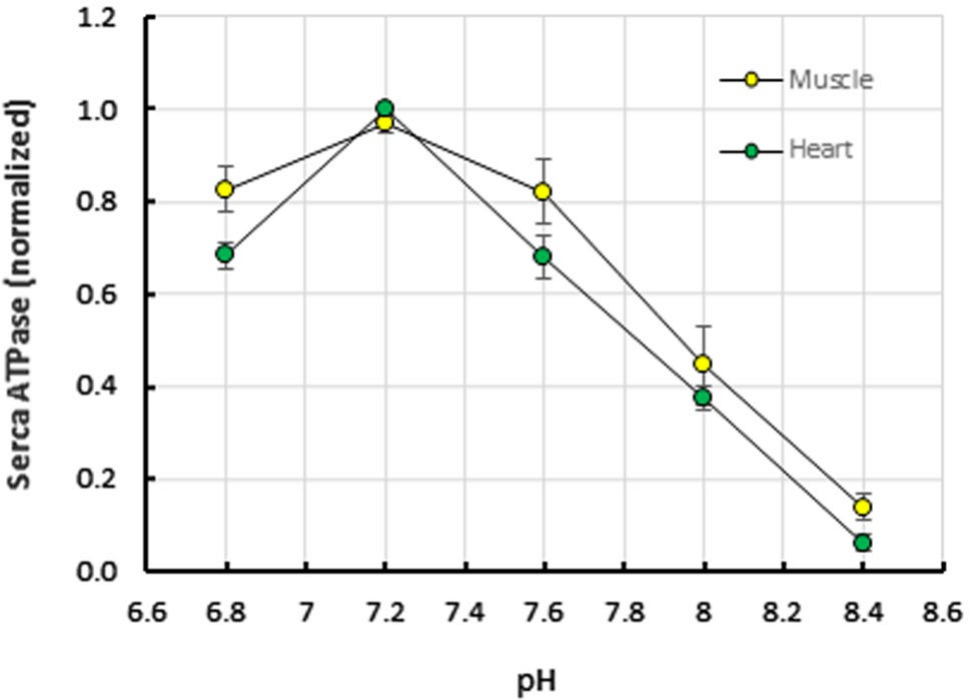
pH dependence of SERCA activity in heart ventricle and dorsolateral myotomal muscle of the brown trout acclimated at + 3 °C (c.a.) or + 13 °C (w.a.). The experiments were conducted at + 23 °C. The results are means ± SEM of 5 and 8 homogenates (= fish) for heart and muscle, respectively. SERCA activity was measured as Ca^2+^-ATPase activity inhibited by either cyclopiazonic acid (CPA, 20 µM) or thapsigargin (TG, 20 µM). Activities were normalized to maximum activity, regardless of the concentration at which it occurred. pH dependence of SERCA activity was statistically significant (two-way ANOVA, *F* = 68.61, *p* = 0.000) and there was a difference between heart and muscle (two-way ANOVA, *F* = 4,55, *p* = 0.037)

CPA- and TG-sensitive Ca^2+^-ATPase activities of heart and muscle were measured in the range of 6.8–8.4 pH units at + 23 °C (Fig. 1). The results are combined data from experiments using TG or CPA as an inhibitor and preparations of both c.a. and w.a. fish. The optimum pH for both preparations was 7.2. At pH values higher than 7.2, the activities declined in almost liner manner (two-way ANOVA, *F* = 68.61, *p* = 0.000) with slight differences between heart and muscle SERCA (two-way ANOVA, *F* = 4.55, *p* = 0.037). In subsequent experiments, the pH of the incubation medium was adjusted to 7.2 at room temperature and allowed to change in alpha-stat manner at other experimental temperatures. The experimentally determined pH values at + 3, + 13, + 23 and + 33 °C were 7.39, 7.28, 7.20 and 7.11, respectively. These values mean that the calculated free Ca^2+^ concentration was 5.3, 6.7, 8.3 and 10.5 µM at + 3, + 13, + 23 and + 33 °C, respectively.

### Inhibition of Ca^2+^-ATPase activity by CPA and TG

**Fig. 2:**
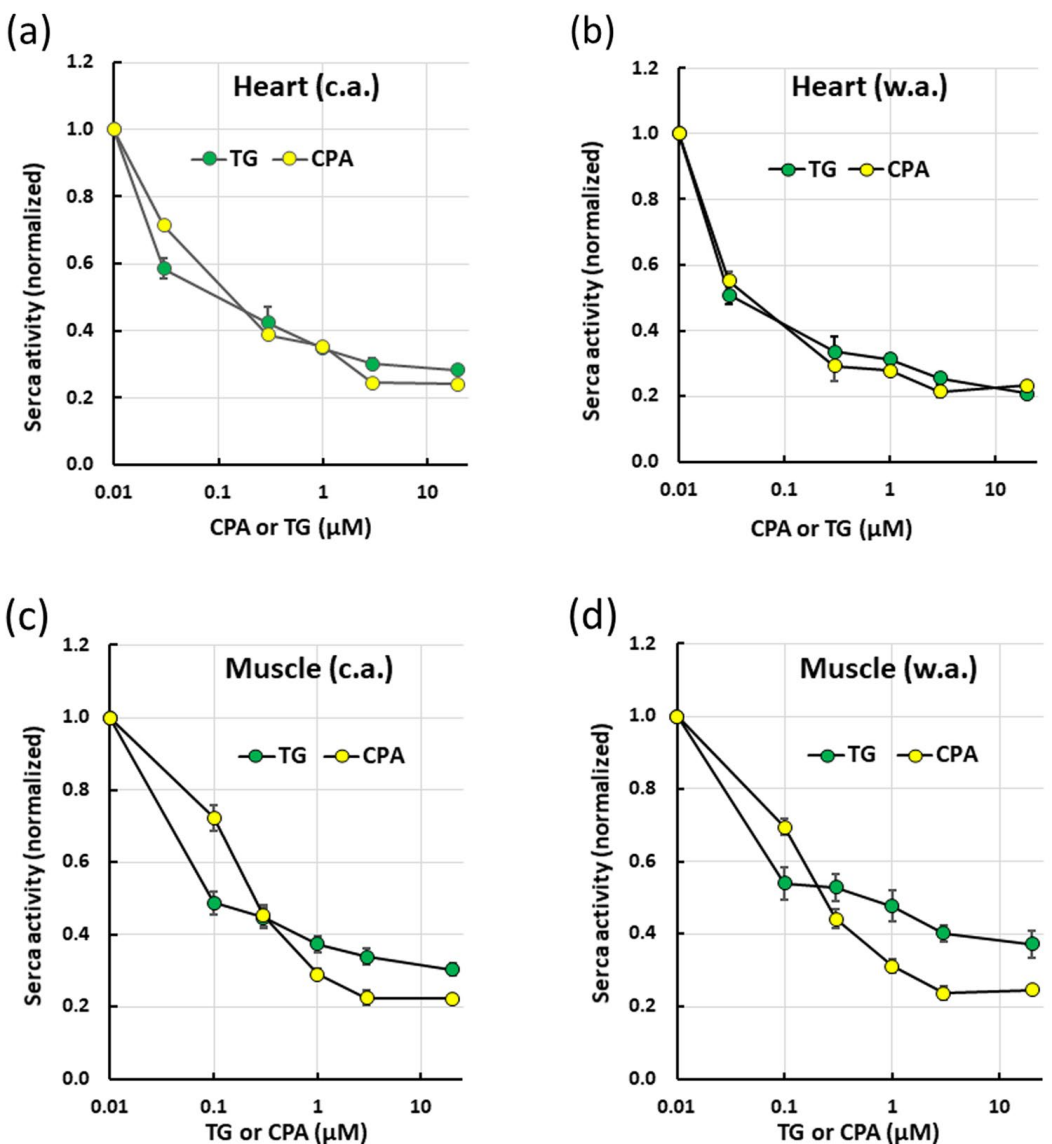
Concentration-dependent inhibition of heart ventricle (**a**, **b**) and dorsolateral myotomal muscle (**c**, **d**) SERCA by thapsigargin (TG) and cyclopiazonic acid (CPA). The experiments were carried out in preparations from both cold-acclimated (+ 3 °C, c.a.) (**a**, **c**) and warm-acclimated (+ 13 °C, w.a.) (**b**, **d**) brown trout. The experiments were made at + 23 °C and at pH 7.2. The results are means ± SEM of 5–8 homogenates (= fish) for the muscle and 3–4 homogenates (= fish) for the heart. The activities were normalized to the control value in the absence of blockers. TG and CPA significantly inhibited heart SERCA in w.a. (two-way ANOVA, *F* = 31.32, *p* < 0.001) and c.a fish (two-way ANOVA, *F* = 229.08, *p* = 0.001) and muscle SERCA in w.a. (two-way ANOVA, *F* = 148.77, *p* < 0.001) and c.a. (two-way ANOVA, *F* = 320.57, *p* < 0.001) fish. Effects of CPA and TG were statistically different in w.a. muscle SERCA (two-way ANOVA, *F* = 13.58, *p* < 0.001)

To find the best way to specifically inhibit SERCA, the efficacy of CPA and TG as inhibitors of muscle and heart Ca^2+^-ATPase were examined at + 23 °C (Fig. 2). TG and CPA were equally effective in inhibiting the Ca^2+^-ATPase of the heart in both c.a (two-way ANOVA, *F* = 0.29, *p* = 0.593) and w.a (two-way ANOVA, *F* = 0.000, *p* = 0.998) trout (Fig. 2a, b). The maximal inhibition was attained at 5 and 20 µM for CPA and TG, respectively. At 20 µM the inhibition of the heart, Ca^2+^-ATPase was 76% (c.a.) and 77% (w.a.) for CPA and 72% (w.a.) and 79% (c.a.) for TG. In contrast to the heart Ca^2+^-ATPase, in the muscle preparations, the inhibition potency of TG and CPA varied between drug concentrations and these were statistically significant for w.a. (two-way ANOVA, *F* = 13.59, *p* < 0.001) but not in c.a. preparations (two-way ANOVA, *F* = 0.223, *p* = 0.638). However, the responses of w.a. and c.a. preparations were strikingly similar. At the concentration of 0.1 µM, TG was a stronger inhibitor than CPA for the muscle Ca^2+^-ATPase (Fig. 2c, d). Conversely, at the concentrations of 1, 5 and 20 µM CPA was a more effective inhibitor of the muscle Ca^2+^-ATPase than TG (Fig. 2c, d). At the concentration of 20 µM, CPA inhibited 78% and 75% of the total Ca^2+^-dependent ATPase activity in c.a. and w.a. trout, respectively, while 20 µM TG inhibited 63% and 70% of the enzyme activity in w.a. and c.a. trout, respectively. Since CPA inhibited muscle and heart Ca^2+^-ATPases of w.a. and c.a. fish to the same extent, 20 µM CPA was used in all subsequent experiments. In conclusion, 72–79% of the Ca^2+^-activated ATPase is due to the SERCA in both tissues. Therefore, from now on, the term "SERCA" will be used to refer to this CPA- and TG-sensitive Ca^2+^-ATPase.

### Temperature dependence of SERCA

**Fig. 3:**
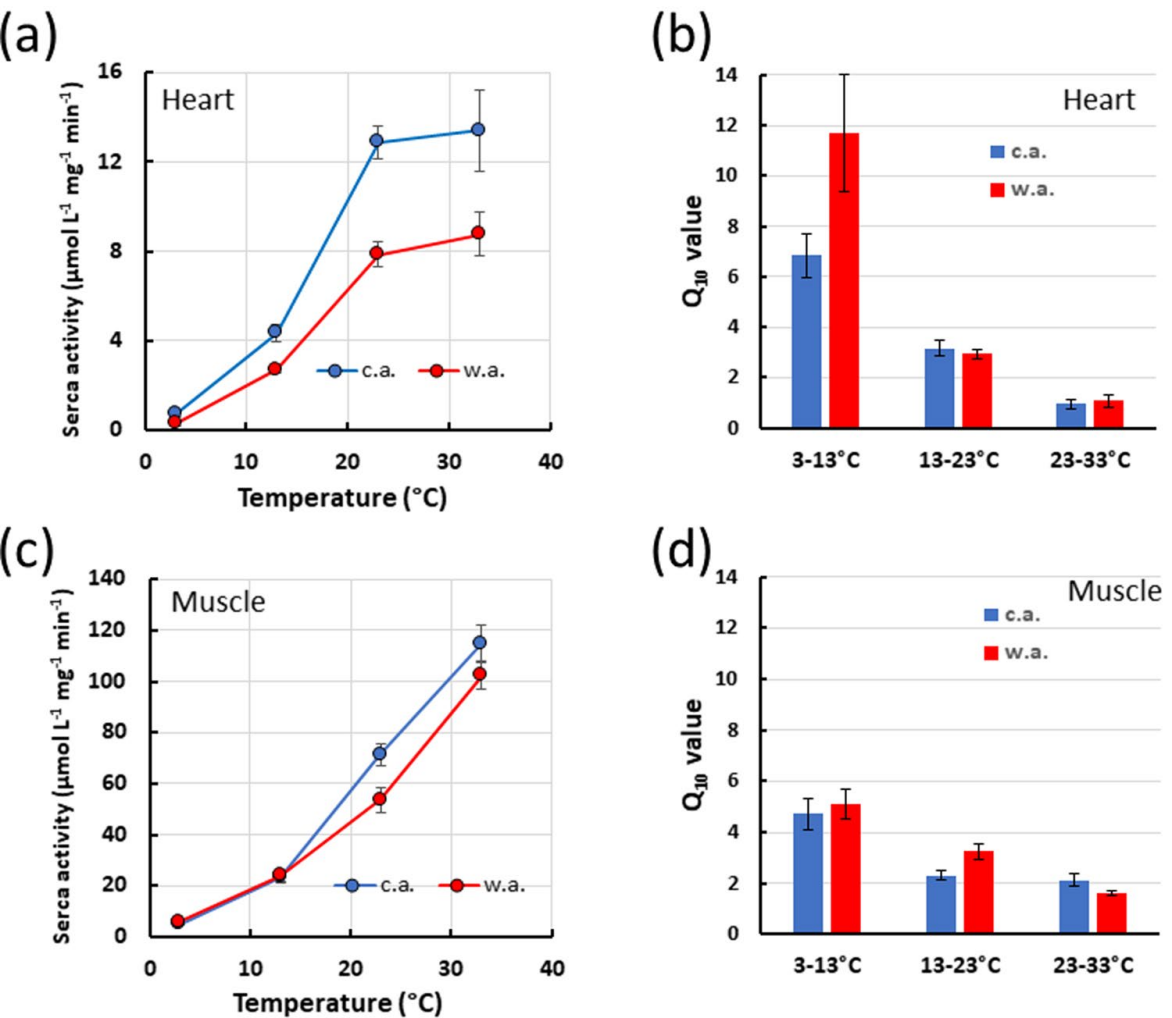
Temperature dependence of SERCA activity in heart ventricle (**a**, **b**) and dorsolateral myotomal muscle (**c**, **d**) from cold- (+ 3 °C, c.a.) and warm- (+ 13 °C, w.a.) acclimated brown trout. Panels **a** and **c** show SERCA activity (µmols of inorganic phosphate liberated by mg tissue wet weight in 1 min) as a function of temperature. Panels **b** and **d** show *Q*_10_ values of SERCA. The results are means ± SEM of 11 and 8 preparations for muscle and heart, respectively. Temperature significantly affected SERCA activity in both heart (two-way ANOVA, *F* = 82.67, *p* < 0.001) and muscle (two-way ANOVA, *F* = 249.48, *p* < 0.001) and the activities were different between acclimation groups both for heart (two-way ANOVA, *F* = 26.33, *p* < 0.001) and muscle (two-way ANOVA, *F* = 6.21, *p* = 0.015) SERCA

Activity of SERCA was studied in temperatures from + 3  to + 33 °C (Fig. 3). The activity of the heart SERCA was significantly higher in c.a. than w.a. trout (two-way ANOVA, *F* = 26.33, *p* = 0.000). At lower temperature (+ 3–+ 23 °C), SERCA activity increased strongly, while above + 23 °C the rate of activity appeared to level off (Fig. 3a). Temperature dependence (*Q*_10_ value) of the heart SERCA was highest between + 3 °C and +13 °C (6.8 ± 0.8 and 11.7 ± 2.31 for c.a. and w.a. trout, respectively) and decreased at higher temperatures without differences between acclimation groups (two-way ANOVA, *F* = 2.71, *p* = 0.107) (Fig. 3b). Arrhenius plots of the heart SERCA (not shown) were linear (*r*^2^ = 0.97–0.98) between + 3 and + 23 °C with *E*_a_ values of 98.4 kJ mol^−1^ (23.5 kcal mol^−1^) and 110.7 kJ mol^−1^ (26.5 kcal mol^−1^) for c.a. and w.a heart, respectively. SERCA activity of the muscle increased almost in exponential manner with increasing temperature (Fig. 3c). The muscle SERCA activity of the c.a. trout was slightly higher than that of the w.a. trout (two-way ANOVA, *F* = 6.21, *p* = 0.015). In contrast to the heart SERCA, there were no signs of thermal inactivation of the muscle SERCA at + 33 °C. Temperature dependence (*Q*_10_ value) of the muscle SERCA was highest between + 3 and + 13 °C (4.7 ± 0.4 and 5.1 ± 0.6 for c.a. and w.a. trout, respectively) and decreased at higher temperatures without differences between acclimation temperatures (two-way ANOVA, *F* = 0.813, *p* = 0.371). Arrhenius plots of the muscle SERCA (not shown) were linear (*r*^2^ = 0.98) throughout the whole temperature range with *E*_a_ values of 73.8 kJ mol^−1^ (17.7 kcal mol^−1^) and 65.5 kJ mol^−1^ (15.7 kcal mol^−1^) for c.a. and w.a muscle, respectively.

**Fig. 4:**
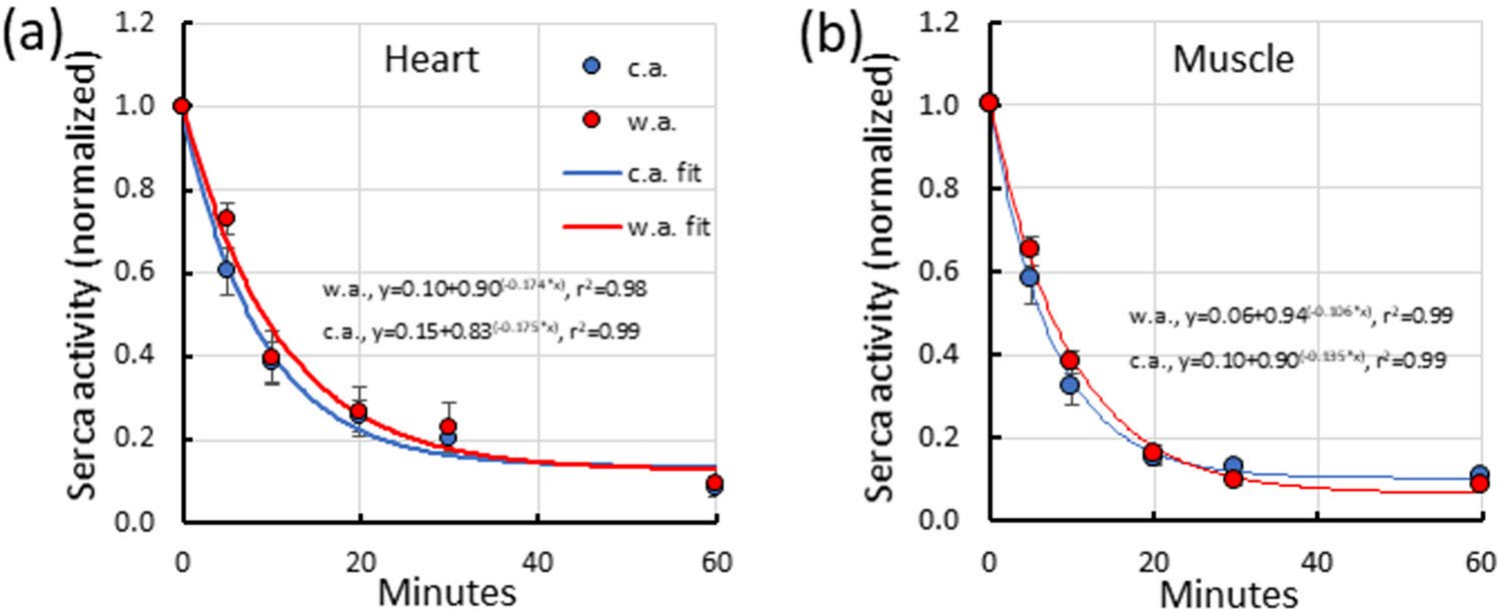
The rate of thermal inactivation of heart ventricle (**a**) and dorsolateral myotomal muscle (**b**) SERCA activity of cold-acclimated (+ 3 °C, c.a.) and warm-acclimated (+ 13 °C, w.a.) brown trout. The results are means ± SEM of 11 and 8 preparations for muscle and heart, respectively. The activities were normalized to the value of the untreated preparations. The rates of inactivation (*τ*) were not different between w.a. and c.a. fish either in heart (Mann–Whitney, *p* = 0.11) or muscle (Mann–Whitney, *p* = 0.16)

The rate of thermal inactivation was studied by exposing the heart SERCA to + 35 °C and the muscle SERCA to + 40 °C for various periods of time after which the activity was measured at + 25 °C for 15 min (Fig. 4). The rate of inactivation followed a monoexponential pattern with time constants of 9.41 ± 1.76 and 11.20 ± 2.28 min for the heart SERCA of c.a. and w.a. fish, respectively (Mann–Whitney, *p* = 0.11) (Fig. 4a). The respective time constants of inactivation for the muscle SERCA were 7.77 ± 1.36 and 9.56 ± 0.79 min for c.a. and w.a. trout, respectively (Mann–Whitney, *p* = 0.16) (Fig. 4b). These analyses show that there is no difference in the rate of thermal inactivation between c.a. and w.a. trout either for muscle or heart SERCA. On the other hand, the muscle SERCA requires higher temperatures for inactivation than the heart SERCA.

## Discussion

### Methodological considerations

The use of unfractionated homogenates has some advantages in comparison to purified SR microsomes in the quantitative measurement of the SERCA activity. First, the unfractionated homogenate contains all the SR membrane present in the tissue and eliminates the variation in SR yield between tissues and species due to purification process. Second, it enables measurement of SERCA activity from tissues with relatively low cellular SR content like many fish hearts and in small fish with a low tissue yield.

On the other hand, the unfractionated homogenate contains several ATPase enzymes and, therefore, one must rely on specific inhibitors to measure the desired ATPase activity. A range of concentrations of the specific inhibitors of the SERCA, TG and CPA, were examined to determine the drug concentration for the maximal inhibition of the activity (Inesi and Sagara 1994). For the heart homogenates, TG and CPA were equally effective in inhibiting SERCA without any difference between drug concentrations. For the muscle homogenates, TG was a stronger inhibitor at the concentration of 0.1 µM, while at the concentrations of 3, 5 and 20 µM, CPA was more effective. TG is known to have a higher affinity than CPA for the SERCA (Ma et al. 1999). This probably explains the strong inhibitory effect of TG at low concentrations. On the other hand, TG tends to bind nonspecifically to other muscle proteins (Du et al. 1994), and may, therefore, lose some of its potency at higher concentrations, particularly in muscle homogenates with almost an order of magnitude more SERCA binding sites in comparison to heart homogenates. Based on these findings, CPA was a better choice as an inhibitor of the SERCA in homogenates because, at the concentration of 20 µM, it inhibits both heart and muscle enzyme to similar extent and without difference between acclimation groups.

### Comparison of heart and muscle SERCA activities

The speed of muscle relaxation is closely related to the rate of Ca^2+^ sequestration by the SR (Cavagna et al. 2000; Rosati et al. 2008). Ca^2+^ uptake into the SR occurs by the activity of the SERCA with the rate being determined by the number of pump units and the catalytic rate of each SERCA isoform (Cavagna et al. 2000). Fast twitch skeletal muscle fibres have a higher SERCA pump density and higher rate of Ca^2+^ uptake than slow twitch fibres or cardiac myocytes. Furthermore, the muscle SERCA1 isoform has higher intrinsic turnover rate than the heart SERCA2 isoform (Lytton et al. 1992; Sumbilla et al. 1999; Cavagna et al. 2000). In the brown trout homogenates, the activity of the muscle SERCA was 5–9 times higher than that of the heart SERCA, which is consistent with the relative importance of SR Ca^2+^ uptake in relaxation of vertebrate skeletal fibers and cardiac muscle cells. Indeed, the muscle/heart SERCA activity ratio in brown trout is similar to the ratio of SERCA protein isoforms in fast twitch muscle and heart ventricle of rat (Wu and Lytton 1993). These findings suggest that the present method of SERCA determination is suitable for comparing the activity of SERCA between heart and muscle homogenates in fish and may also be useful more generally in comparing SERCA activity between different species and tissues.

### Effect of temperature acclimation on SERCA activity

Acclimation to cold increased the specific activity of the heart SERCA of the brown trout. However, the thermal compensation was only partial, since the activity of the w.a. brown trout at + 13 °C was higher than the activity of c.a. brown trout at + 3 °C. At the common experimental temperatures (+13–+ 33 °C), the heart SERCA activity was 47–64% higher in c.a. than w.a. brown trout. The SERCA activity seems to be fairly well tuned to the maximal heart rate of the fish which is about 62% higher in c.a. than w.a. brown trout (Vornanen et al. 2014; Haverinen et al. 2017). The temperature response of the SERCA in the heart of brown trout is similar to that of the rainbow trout (*O. mykiss*), whose TG-sensitive Ca^2+^ uptake rate was doubled in the cold-acclimated (+ 4 °C) fish (Aho and Vornanen 1998). Obviously, the improved Ca^2+^ sequestration by the SR is part of the cellular machinery, which enhances contraction kinetics and allows compensatory increase in heart rate in the cold-acclimated fish (Bowler and Tirri 1990; Keen et al. 1994; Aho and Vornanen 1999, 2001; Vornanen et al. 2002; Klaiman et al. 2011; Shiels et al. 2011). In principle, the cold-induced increase in the activity of heart SERCA could be accomplished by temperature-induced changes in the lipid composition of the SR membrane, increase in the number of SERCA pump units or increase in the turnover rate of the enzyme due to changes in phospholamban/SERCA ratio or increased phosphorylation level of the phospholamban (Ushio and Watabe 1993; Ushio et al. 1997; Sumbilla et al. 1999; Vornanen et al. 1999; MacLennan and Kranias 2003). Cold-induced increase in SERCA transcripts of both atrium and ventricle has been reported for rainbow trout and blue-fin tuna (*Thunnus orientalis*) hearts (Korajoki and Vornanen 2012; Jayasundara et al. 2013). At the protein level, SERCA has been found to be upregulated in the atrium of the cold-acclimated (+ 4 °C) rainbow trout and in atrium and ventricle of the cold-acclimated (+ 4 °C) burbot (*L. lota*) (Korajoki and Vornanen 2012, 2013). Furthermore, in the rainbow trout, acclimation to cold reduced the atrial phospholamban/SERCA ratio thus supporting higher SERCA activity in the cold (Korajoki and Vornanen 2012). Collectively, these findings indicate that the activity of the heart SERCA is enhanced in the cold-acclimated fish by increase in the number of pump units and reduction in phospholamban/SERCA ratio, while the role of membrane lipids remains open. The unfractionated homogenates contain all necessary molecular entities needed for SERCA function. Therefore, this preparation may provide an opportunity to further investigate the role of phospholamban in thermal responses of SERCA. Experiments in zebrafish suggest that thyroid hormones are crucial in supporting high SERCA activity and high resting and maximum heart rates in cold-acclimated (+ 18 °C) individuals of this species (Little and Seebacher 2014). However, in the rainbow trout acclimated at + 13 °C thyroid state did not affect ryanodine sensitivity of force generation, force restitution or kinetics of contraction, even though the heart rate was changed (Tiitu and Vornanen 2003). This suggest that the role of thyroid hormones in regulation of SERCA activity may vary between species and depending on the acclimation temperature of the fish.

Acclimation to cold induced a slight increase in the activity of muscle SERCA of the brown trout (12% and 33% at + 23 °C and + 33 °C, respectively). The response of SERCA activity to temperature acclimation in brown trout muscle is qualitatively similar to that of the common carp (*Cyprinus carpio*) but less pronounced. In the myotomal muscle of the common carp cold-acclimation (+  8 °C) induced a 60% increase in the SERCA activity relative to warm-acclimated (+  20 °C) fish (Fleming et al. 1990). Ushio and Watabe found twice higher SERCA activity in the myotomal muscle of the cold acclimated (+ 10 °C) than warm acclimated (+  30 °C) common carp when measured at the common experimental temperature (Ushio and Watabe 1993). Thus, the less pronounced effect of acclimation in brown trout may be due to smaller difference between the acclimation temperatures. The higher SERCA activity of the cold-acclimated carp was associated with increased fluidity of SR membranes which may enable higher catalytic activity of the enzyme (Ushio and Watabe 1993). Similar to the heart SERCA cold-induced increase in the activity of the muscle SERCA is probably contributing to the faster isometric contraction kinetics of the cold-acclimated myotomal muscle in fish (Fleming et al. 1990; Hwang et al. 1991). In the zebrafish muscle, acclimation to cold (+ 18 °C) does not affect SERCA activity, but thyroid hormones seems to be needed to maintain SERCA activity in the cold-acclimated fish (Little and Seebacher 2013).

### Temperature tolerance of SERCA

The main objective of the present study was to test whether the SERCA of the brown trout heart might be involved in thermal failure of the heart and, therefore, potentially involved in setting the thermal tolerance limits of the whole animal. To this end, two working hypotheses were put forward, both of which should be valid if the SERCA plays a crucial role in determining temperature tolerance of the heart and the fish. However, only one of these conditions was found to be correct by the experimental observations.

The heart SERCA was more sensitive to high temperatures than the muscle SERCA, which is consistent with our first working hypothesis predicting a lower heat tolerance of the heart enzyme relative to that of the muscle enzyme. Activity of the muscle SERCA increased with warming up to + 33 °C, much above the upper incipient lethal temperature of the brown trout (+ 22–+ 25 °C) (Elliott and Elliott 2010), without any signs of inactivation. In the studied temperature range of + 3–+ 33 °C, Arrhenius plots of the muscle SERCA were linear and similar to that of several other teleost species (McArdle and Johnston 1980; Vrbjar et al. 1990; Chini et al. 1993; Feher et al. 1998). These findings strongly suggest that the SERCA is capable for fast muscle relaxation throughout the upper thermal tolerance range of the brown trout. In contrast, the Arrhenius plots of the heart SERCA were linear only between + 3 and + 23 °C, after which the activity increased only marginally. Furthermore, the heart SERCA was inactivated at + 35 °C as fast as the muscle SERCA at + 40 °C. These findings indicate that the heat tolerance of the heart SERCA is substantially lower than that of the muscle SERCA. Nevertheless, the activity of the heart SERCA increased exponentially to the upper incipient lethal temperature of the brown trout and did not decrease even at + 33 °C. This suggests that the peak activity of the heart SERCA is achieved somewhere between + 23 and + 33 °C. Although more sensitive to thermal inactivation than the muscle SERCA the heart SERCA seems to be sufficiently heat tolerant to accomplish relaxation of the brown trout ventricle at habitat temperatures experienced by the fish (Elliott and Elliott 2010).

Second, if the SR Ca^2+^ uptake would be the limiting factor for contraction rate of the ventricle, high temperature tolerance of the heart SERCA should vary with the acclimation temperature (Bowler 1981, 2018). Contrary to this hypothesis heat tolerance of the heart SERCA was almost identical in w.a. and c.a. brown trout. The rate of thermal inactivation, *Q*_10_ values of the activity and activation energy were similar for c.a. and w.a. SERCA. This is inconsistent with the documented heat sensitivity of heart rate in the thermally acclimated brown trout. Cold acclimation shifts the breakpoint temperature of heart rate to lower values by almost 8 °C: the breakpoint temperatures for + 2 °C-acclimated and + 12 °C-acclimated fish are + 15.7 °C and + 23.5 °C, respectively (Vornanen et al. 2014; Haverinen et al. 2017). Because the SERCA of the c.a. brown trout tolerates much higher temperatures than the ventricular beating rate, it cannot be the limiting factor for ventricular contraction rate (Haverinen and Vornanen 2020). The present findings are consistent with those from the rainbow trout and coho salmon (*Oncorhynchus kisutch*), whose SERCA maintain high activity throughout the temperature range from + 5 to + 30 °C (Da Silva et al. 2011; Landeira-Fernandez et al. 2012). However, *Q*_10_ values of the brown-trout SERCA seem to be higher than those of the *Oncorhynchus* species (Aho and Vornanen 1998; Hove-Madsen et al. 2001; Da Silva et al. 2011; Landeira-Fernandez et al. 2012). If this difference holds also in vivo conditions, the responses of the brown trout SERCA to acute temperature changes are sharper than in *Oncorhynchus*.

In the sinoatrial pacemaker cells of toad and mammalian hearts, spontaneous Ca^2+^ releases from the SR activate sarcolemmal Na^+^–Ca^2+^-exchange and the generated inward current contributes to pacemaker rate via the Ca^2+^-clock mechanism (Ju and Allen 1999; Lipsius et al. 2001; Monfredi et al. 2013; Yaniv et al. 2015). Consistent with the Ca^2+^-clock mechanism simultaneous application of ryanodine and TG to sinoatrial preparations of the rainbow trout heart reduces their spontaneous beating rate (Haverinen and Vornanen 2007). Notably, the effect is stronger at + 18 °C than at + 11 °C. Because the activity of the SR seems to affect pacemaker rate more strongly at high temperature, it is in principle possible that SERCA is directly contributing to thermal failure of heart rate. However, if it is assumed that the SERCA activity of the pacemaker cells is equally tolerant against high temperature as the SERCA of the ventricle, the high temperature tolerance of the pacemaker rate should not be compromised by the Ca^2+^-clock mechanism.

### Final conclusions

Temperature acclimation does not change high temperature tolerance of the heart SERCA in brown trout suggesting that it is not the limiting factor for thermal tolerance of the ventricular beating rate. The cold-induced upregulation of the activity of the heart SERCA is achieved by a quantitative increase in the pump units and perhaps via the reduced phospholamban/SERCA ratio (Korajoki and Vornanen 2012, 2013). Thus, the SERCA activity of the brown trout heart is adjusted to temperature-induced changes in heart rate mainly by quantitative means without changes isoform composition or thermal tolerance of the pumps.
